# Exploring barriers and facilitators to PrEP use among transgender women in two urban areas: implications for messaging and communication

**DOI:** 10.1186/s12889-021-12425-w

**Published:** 2022-01-06

**Authors:** Sarah Bauerle Bass, Patrick J. Kelly, Jesse Brajuha, Luis Gutierrez-Mock, Kimberly Koester, Paul D’Avanzo, Jae Sevelius

**Affiliations:** 1 Department of Social and Behavioral Sciences, Risk Communication Laboratory, Temple University College of Public Health, Temple University College of Public Health, Risk Communication Laboratory, 1301 Cecil B. Moore Ave., Room 945, Philadelphia, PA 951 USA; 2 Center of Excellence for Transgender Health, University of California San Francisco, San Francisco, CA USA

**Keywords:** HIV prevention, Transgender women, PrEP, Focus group, Communication

## Abstract

**Background:**

Trans women are at increased risk for HIV infection yet are less likely to use pre-exposure prophylaxis (PrEP) medication as a preventive measure. PrEP messaging and marketing has focused on men who have sex with men (MSM) or included trans women as a subset of MSM, ignoring the potential barriers to PrEP use unique to trans women. Little is known about how this group conceptualizes PrEP, what knowledge gaps still exist, and how trans women believe PrEP should be communicated to increase use.

**Methods:**

This qualitative study conducted focus groups (*n* = 5) in Philadelphia and Sacramento with trans women to assess these issues.

**Results:**

Twelve sub-themes were found related to five main domains, including PrEP knowledge, benefits, barriers, community-related considerations, and messaging/marketing. Findings indicate that knowledge of PrEP is still low and beliefs about PrEP’s effects on hormone use persist. Most importantly, participants voiced a demand for culturally appropriate trans-specific messages in HIV prevention interventions and communication.

**Conclusions:**

Without acknowledging specific barriers to PrEP uptake among transgender women separate from those of MSM and incorporating gender affirmation into PrEP education, simply knowing PrEP is available may not motivate trans women to use PrEP. This has important implications for future efforts to communicate about PrEP with trans women.

**Supplementary Information:**

The online version contains supplementary material available at 10.1186/s12889-021-12425-w.

## Background

In the United States transgender women (trans women) are 34 times more likely to be living with human immunodeficiency virus (HIV) than the general population and HIV prevalence among trans women is estimated to be 14% [1, 2]. Importantly, figures that characterize HIV rates among trans women are likely underestimated because the majority of U.S. HIV reporting districts do not systematically capture trans identities when collecting HIV surveillance information [3]. Some trans-identified individuals may also be reluctant to disclose their identity [4, 5].

In the absence of systematized data, empirical studies provide insight into the disparate impact of HIV among trans women. Over the five-year period 2014–2018, diagnoses of HIV among U.S. trans people increased and in 2018, trans people accounted for 2% of incident 2018 HIV diagnoses among all adults and adolescents in the U.S. and six dependent areas [6]. In 2017, the percentage of trans people who were tested for HIV and newly positive was three times the national average [7]. Moreover, data from the National HIV Surveillance System found that of the 2351 trans individuals who were newly diagnosed with HIV from 2009 to 2014, 84% were trans women [4]. A recent prevalence study in seven US cities indicated that 51.2% of trans women in Philadelphia and 41.2% in San Francisco, the areas where this study occurred, were living with HIV [8].

HIV prevalence is highest among trans women of color; a 2019 CDC meta-analysis estimated mean HIV prevalence was 44.2% among African American trans women and 25.8% among Latina trans women compared to 6.7% among White trans women [7]. High levels of stigma and discrimination against trans women [9], which may reduce social support and increase engagement in high-risk behaviors such as survival sex work, may position trans women to be at increased risk for HIV acquisition [10]. One study noted that one in five trans persons reported avoiding seeking medical care because of past negative experiences and transphobia in medical settings [9] and two other studies noted that trans women reported avoiding HIV testing because the settings are not known to be trans-friendly [11, 12].

Pre-exposure prophylaxis (PrEP) is a medication that, when taken as prescribed, reduces the risk of HIV in men who have sex with men (MSM), heterosexual men and women, and injection drug users [13–15]. However, a subanalysis of data from the iPrEx study, the first PrEP clinical trial, showed that among the trans women participants PrEP was less effective, likely due to low uptake and poor adherence [15, 16]. In general, trans women have been overlooked in PrEP research; a 2019 U. S Preventative Services Task Force review found that trans women remain seriously under-enrolled or are aggregated with MSM in clinical trials of effectiveness of oral PrEP [17]. Studies of unique barriers to PrEP uptake among trans women have highlighted low levels of knowledge about PrEP, medical mistrust, concerns about potential drug interactions with hormones, and PrEP marketing that centers on gay men [18, 19]. However, these studies have occurred in small geographic regions and investigations of PrEP perceptions and experiences among trans women across geographic areas are needed as PrEP availability increases.

While some studies have investigated the unique and complex barriers to PrEP uptake and adherence among trans women [18, 20–23], more information to inform specific ways to communicate about PrEP within the context of the needs of trans women specifically, as they are disaggregated from MSM, is needed. While culturally relevant messages and interventions could be powerful tools in helping increase PrEP awareness among at-risk HIV-negative trans women and address barriers to PrEP initiation, the development of these interventions is hindered by limited understanding of how best to communicate about PrEP among trans women. Specifically, targeted interventions must acknowledge and address the specific barriers to PrEP use in trans women that use language, images and content that are grounded in the community, and that include understanding of stigma and discrimination [24, 25]. Because of this, we used a systematic formative research process adopting the overarching principles of the Social Marketing Framework, which emphasizes an audience-centered consumer orientation and the belief that communication interventions must be grounded in the experience and perceptions of the target group [26]. Thus, this study sought to qualitatively investigate trans women’s current knowledge, experiences, and perceived barriers and facilitators related to PrEP, as well as input on communication, messaging, and marketing needs, to inform tailored messaging that could be embedded in communication interventions for PrEP uptake and adherence in two urban locations.

## Methods

### Study location, population, and recruitment

We conducted five focus groups at centrally located trans-friendly organizations including community-based agencies or health clinics in Philadelphia, PA (*n* = 3; *n* = 20) and Sacramento, CA (n = 2; *n* = 14) from June to November 2017. Potential participants were recruited through social media posts, flyers at local trans-friendly organizations, and snowball sampling methods in which women were recruited by other trans women to participate. Participants completed a written consent form and were verbally screened for eligibility prior to participation. Eligible persons included those who self-reported HIV negative status, were 18 years of age or older, could speak and understand English and who identified as a woman, trans woman, or transfeminine and were assigned male sex at birth. We did not exclude people by PrEP status as we wanted a variety of opinions about how PrEP is perceived of in the trans women community.

Eligible participants were invited to participate in a focus group to discuss knowledge of PrEP and perceived barriers, benefits, and concerns about PrEP among trans women. Focus group methodology was chosen because we wanted participants to be able to exchange viewpoints and talk about their experiences with PrEP and how it is perceived of in their community [27], allowing for active participation and discussion. Groups lasted approximately 1 h and were moderated by members of the study team who have significant experience in qualitative methods [18, 28–30]. Moderators all have degrees in public health or health psychology and were accompanied with members of the trans community in the two locations.

All groups included a description of the study, why we were doing it, and how the moderator was involved in the research. Sessions were audio recorded and transcribed verbatim by individuals on the research team. Observers also took fieldnotes to note anything that would not be evident through the audio recordings. Participants received a $25 gift card for their participation. All procedures were approved by the Institutional Review Boards at Temple University (#24073) and the University of California, San Francisco (#17–21,717).

### Instruments

Focus group participants completed a sociodemographic questionnaire (Additional file 1) which assessed: race, age, educational attainment, employment status, housing security, income and source, financial security, history of homelessness, insurance status and insurance source. Additionally, participants were asked if they had heard about PrEP from a doctor, family member, friend, or someone in the community, and whether or not they had ever used PrEP. If they indicated they had used PrEP, they were also asked how long they had taken it or if they were still taking it.

A focus group moderator’s guide (Additional file 2) was developed based on previous research by the investigators and experience working with trans women and PrEP [18, 31–35], and further refined by consulting medical experts who provide PrEP and healthcare to trans women. Topics addressed included: experiences taking or hearing about PrEP, personal and structural barriers to taking PrEP, stigmas associated with taking PrEP, perceived benefits and risks of taking PrEP, and input on best ways to communicate about PrEP to trans women.

### Analysis

Descriptive statistics (e.g., means, standard deviations, frequencies) were used to summarize demographic information of focus group participants. Chi-square tests were used to test for associations between categorical variables and study sites. Phi and Cramer’s v were assessed in cases where cell sample size was < 5. A *p-value* threshold of 0.05 was used to determine statistical significance. Statistical analysis was completed using for Statistical Package for Social Sciences (SPSS) version 25.0.

Focus group transcriptions were uploaded to Dedoose, an online mixed-methods analysis program, to facilitate qualitative analysis. Analytic efforts were guided by the research question: “How do trans women think about PrEP and how do they want to be communicated to about PrEP”. Interpretation of the data was based on a phenomenology approach in which study of an individual or group’s (i.e., trans women in two urban areas) lived experiences and knowledge about something [i.e. PrEP] are shared [36]. The analysis was not guided by a health behavior theory per se because results were meant to inform message development, not behavior outcomes. Instead, the analysis focused on similarities and differences within the groups and between cities to understand how nuanced experiences could potentially be addressed in targeted messaging. Thematic analysis was thus conducted using the Krueger method: familiarization, identifying a thematic framework, indexing, charting, mapping and interpretation [37]. An initial codebook was developed consisting of deductive codes extracted from the moderator’s guide and further refined as transcripts were independently read by two study team members. This initial codebook continued to be refined through the addition of inductively created codes. Once the team reached consensus on a final set of codes, two analysts served as either a primary or secondary coder. Discrepancies in code application were resolved through discussion until 100% consensus was reached. Code excerpts were grouped by thematic similarity and summarized.

## Results

### Demographic characteristics

Focus groups ranged in size from 3 to 10 participants (mean = 7). Demographics were similar by site (see Table 1), although the mean age in Philadelphia (46) was higher than Sacramento (34). Sacramento had more participants who identified as Latina/x (43% vs. 5%, *p* = .01). More participants in Philadelphia (60% compared to 29%) had a high school education or below. In both locations, over 60% said they had experienced homelessness at some point. Specific characteristics by each focus group are also available (Additional File 3).

**Table 1:** Demographic Characteristics of Participants by Location

Characteristic	Philadelphia (***n*** = 20)	Sacramento (***n*** = 14)	Total (***N*** = 34)	***p*** ^†^
**Age,** mean (SD)^a^	46.8 (26.8)	34.14 (12.4)	36.03 (14.3)	.075
**Race,** N(%)^b^				
African American	8 (40)	7 (50)	15 (44)	0.56
White	8 (40)	6 (43)	14 (41)	0.87
Latinx	1 (5)	6 (43)	7 (21)	.01
All other races	3 (15)	1 (7)	4 (12)	0.63
**Gender Identity,** N(%)^b^				
Female	2 (10)	5 (36)	7 (21)	0.097
Transwoman	18 (90)	10 (71)	28 (82)	0.162
Queer	1 (5)	1 (7)	2 (6)	1.0
Additional	0	1 (7)	1 (2.9)	0.42
**Educational Attainment,** N(%)				.071
High school and below	12 (60)	4 (29)	15 (44)	
Some college and above	8 (40)	10 (71)	19 (56)	
**Homelessness Experienced,** N (%)	12 (60)	8 (67)	20 (63)	.722

^†^Where contingency tables report cells with expected values < 5, Fisher’s Exact significance is reported

^a^Includes only people who disclosed their age (*n* = 31)

^b^Category is not exclusive

The majority of participants in both Philadelphia (70%) and Sacramento (79%) had heard about PrEP from a doctor or other healthcare provider. Despite this, no Sacramento participants reported previous PrEP use as compared to just over half (55%) of Philadelphia participants (*p* < .000) (Table 2).

**Table 2:** PrEP Information and Use by Location

	Philadelphia (***n*** = 20)	Sacramento (***n*** = 14)	Total (***N*** = 34)	p
**Ever Heard of PrEP from Doctor/Healthcare Provider,** N(%)				.697
Yes	15 (75)	11 (78.6)	7 (20.6)	
No	4 (20)	3 (21.4)	26 (76.5)	
Don’t Know	1 (5)	–	1 (2.9)	
**Ever Heard of PrEP from family, friend, someone in Community, N(%)**				.246
Yes	12 (60)	12 (85.7)	24 (70.6)	
No	7 (35)	2 (14.3)	9 (26.5)	
Don’t Know	1 (5)	–	1 (2.9)	
**Ever used PrEP,** N(%)				.000
Yes	11 (57.9)	0	11 (33.3)	
No	8 (42.1)	14 (100)	14 (66.7)	

### Focus group results

We identified five key domains of inquiry. These included: 1. Knowledge about PrEP (sub topics: PrEP knowledge and effectiveness, PrEP resistance, STI non-protection, pill taking; difference between PEP and PrEP); 2. Sexual behavior (sub topics: who should take PrEP, sex work, condom use); 3. Trans community (sub topics: Importance of community, HIV stigma and PrEP); 4. Healthcare experiences (sub topic: access to care); and, 5. PrEP marketing (sub topics: reaching “hard to reach”, concerns and suggestions). Based on these key domains, four overall themes emerged: 1. Continued lack of knowledge about PrEP and its use; 2. Persistent structural and personal barriers to PrEP use; 3. Beliefs about how PrEP should be integrated into sexual behavior; 4. The importance of the trans community in promoting and communicating about PrEP use. Sample quotes by theme are presented in additional file 4.

#### Continued lack of PrEP knowledge and use

While most participants understood PrEP to be a daily medication that protects people who are HIV-negative from contracting HIV, none of the Sacramento participants and only roughly half of the Philadelphia participants had ever taken PrEP and a number of misconceptions about PrEP were uncovered. A Philadelphia participant (focus group [FG] 1) said: *“I heard that it prevents the HIV spread. If you take it, you start taking it, not like you don’t even take it night you had sex and then wake up thinking you not gonna get it, you have to like “prep” your system for it.”* Participants in both locations understood that while PrEP is highly effective, there are limitations to its use. However, Philadelphia participants were concerned about resistance because the medication is the same as the anti-retroviral that those who have HIV take. This belief led many to believe that taking PrEP could cause you to be resistant should you become infected with HIV and a reason to not take PrEP. One participant (FG 3) said, *“... If you’re on HIV meds and you’re not taking your regimen properly for whatever reason whether it’s homeless, you face some type of homeless, or you just cannot get to your medication, what ends up doing is that your body builds a resistance up to that medication.”* This sentiment was not expressed in Sacramento.

Knowledge of and experiences with post-exposure prophylaxis (PEP) played a role in participant perceptions and support of PrEP as something they might incorporate into their own lives. For example, a Sacramento participant expressed a hypothetical preference for the convenience of PEP because she believed it would minimize PrEP pill burden and be less expensive; she stated “*PEP would probably be more popular because one, if you need it, you can go and get it, like “Oh I just, you know, I need it“ and it’s not like I’m taking it every day for what if, and that’s wasting hella money, so I’d probably most likely do PEP.”*

For some, the preference was a matter of convenience. But lack of knowledge about the challenges of taking PEP coupled with a disinterest in taking PrEP swayed individuals to prefer PEP. However, some pushed back against the concept of PEP as a superior HIV prevention strategy as in the case of a Sacramento participant (FG 4) who said: “… *I think that PrEP is like the peace of mind like, “Oh my god, I might have slipped up last night, but I took this medication so my body is gonna be able to fight it“ and then the PEP is like, “Dammit I slipped and let’s see what’s gonna happen here.”*

#### Persistent structural and personal barriers to PrEP use

Barriers related to PrEP use continue to be both personal (feelings about hormone interaction and pill burden; perceived HIV stigma) and structural (negative healthcare experiences, community level HIV stigma). There were differing opinions about how easy PrEP might be for participants to take. For those with chaotic lives or who were already taking other medications, adding another pill was seen as prohibitive. A Sacramento participant (FG 4) talked about the burden of taking daily hormones along with PrEP. She said, “*So all that plus your daily hormones? That’s like eight pills. That’s a lot”.* Even so, others who acknowledged the burdensomeness of a daily pill felt that despite their hormone replacement therapy regimen, they could integrate PrEP into their daily routine. The ease and utility of taking PrEP was compared to taking a daily vitamin or oral contraceptives. A Sacramento participant (FG 4) said, “*I always explain it like PrEP is the birth control pill for HIV”.*

Participants in Philadelphia talked about HIV stigma as a common community attitude and that taking PrEP could exacerbate this feeling. This may not have been an issue in Sacramento since none of the participants had taken PrEP and are less likely to have encountered experiences where they were confronted with HIV stigma related to PrEP use. In Philadelphia many discussed being afraid that someone would assume they had HIV if they saw PrEP medication. One participant (FG 3) said: “*Imagine if someone who is not aware or knowledgeable about PrEP goes to your medicine cabinet, sees Truvada. Never heard of this medication. If you like me, you gonna Google … And they see Truvada. The first thing they see is HIV, you know people minds get all fuzzy ‘oh shit*.”

Interactions between trans women and their providers influenced their experiences with and willingness to take and adhere to PrEP. Several participants said that their provider never discussed sex and/or PrEP with them. A Philadelphia participant (FG 1) said, “*It’s never been advertised to me. It’s never been something my doctors talked to me about. Weirdly enough I think the first time I heard about it I was in the hospital for a broken finger and they just do HIV testing there*”. Some individuals also discussed the lack of comfort in discussing sex with their providers, as well as previous or current experiences with transphobia or other forms of discrimination, as barriers to getting PrEP and that this was a significant issue for many trans women. A Sacramento participant (FG 5) said, “*So it’s one more hoop to jump through, one more potential barrier. I mean what if the doctor is judgmental? What if they write stuff in my notes that other doctors can see that would end up hurting me later down the road? I mean, I would think about things like that”*. These fears were vocalized even though both Sacramento and Philadelphia have a number of “trans friendly” medical care services.

#### Beliefs about how PrEP should be integrated into sexual behavior

There were varying opinions regarding how PrEP should be used as HIV prevention, especially in terms of sexual behavior. Participants discussed that those who are frequently engaging in sexual activities were appropriate candidates for PrEP, especially if the sex was casual or if they had relationships with multiple people. A Philadelphia participant (FG 2) said: “*There are people that have occasional sex...I’m actually one of those, where it would be, right, do I need to be on it all the time? Can I get some and know I got a date Friday, two weeks from now, can I start taking it now and then stop?”* A number of participants in Philadelphia raised concerns about the faithfulness of partners and that PrEP may still be needed even if in a monogamous relationship. One (FG 1) said, “*We’re all at risk of HIV because I don’t know who my partner’s doing; you don’t know what your husband’s doing, none of us, none of us”*.

The use of PrEP in serodiscordant relationships was only discussed in the Sacramento focus group discussions. One participant (FG 4) said, “*My partner is positive. His viral load is undetectable but I love him. I can’t say that I wouldn’t just not be with him because of it. So just to know that that option is there”.* This is an important distinction as it allows trans women to think about different scenarios in which PrEP may or may not be used and how the timing of using PrEP may be influenced by relationship status.

Discussions about PrEP and sexual behavior among participants in both locations also frequently revolved around HIV risk associated with sex work. People who engage or previously engaged in survival sex work viewed themselves as appropriate candidates for PrEP. A Sacramento participant (FG 4) said, “*In my early twenties I was a wild child, escorted all types of craziness. At that point in my life, I would have definitely been interested in it because I was afraid of everything that was out there and everything that was going on”.*

Discussions about PrEP were also often linked to condom use. In general, participants in both locations had significant problems with having to use condoms and felt there were several barriers to using them, including their reliability, the need to remember to have one, potential conflicts with sexual partners during intimate situations, or simply not liking to use them. A Philadelphia participant (FG 1) said: “*A lot of my girlfriends don’t always be condom prepared. Like, they have everything else but they don’t have condoms and they’re like “sis you got a condom?”*. This can be exacerbated if the sex is transactional. Participants who engaged in sex work shared that clients frequently request to not use them and offer them more money for condomless sex: “*...Regardless if you’re using a condom or not, condoms pop and you’re with multiple members in one night and … sex work is, for many trans individuals, survival technique, so if somebody’s gonna give me $100 …*” (Philadelphia, FG 1). However, one individual (FG 5) mentioned concern that if clients knew that she or other sex workers were on PrEP they would be more likely to pressure them not to use a condom. She said: “*If the clients know that the person they’re seeing that- the sex worker that they’re on a date with- has been taking PrEP, then they might use that as a way to pressure them not to use condoms. If the sex workers don’t tell them, then they’re still going to face a lot of pressure to not use a condom.”*

These barriers to condom use may motivate some to use PrEP. However, participants also expressed that PrEP may be used as an alternative to condoms rather than an additional tool to preserve health, making them at risk for other sexually transmitted diseases. One Philadelphia participant (FG 1) said: “*People are thinking that this is a magic pill* [PrEP] *and that they don’t have to use condoms and the thing is you still have to stress to people that you still have to use condoms to keep yourself at a lower risk, regardless if you’re taking this one pill a day or not”*.

#### Importance of the trans community in promoting and communicating about PrEP use

Participants in both locations believe that PrEP marketing is flawed. Most participants in Philadelphia reported that they had not previously seen advertisements for PrEP specific to trans women. However, one participant (FG 3) also noted that at least in Philadelphia, HIV prevention including PrEP was discussed in the trans community which was not the case when she lived in the Southern United States. She said, “*Moving to Philadelphia I noticed a big difference when it came to say like STI and HIV prevention … I noticed that like they would give away condoms and they would have PrEP. Like I lived in the South, you couldn’t go somewhere and get a free condom or you didn’t hear about PrEP or anything.”*

Most who indicated they had previously seen information about PrEP reported that the messaging they saw was explicitly tailored to men who have sex with men. This was perceived as trans-exclusionary. As one Sacramento participant (FG 4) said, “*Hey, what about the rest of us?”.* Trans women are often erroneously lumped with men who have sex with men in medical research and are not often disaggregated in government level data. This is evidenced by a Philadelphia participant (FG 2) who said, “*I’m not a male who has sex with males. I am of the trans community … I use she/her, they/them”.*

Individuals who are withdrawn from the transgender community were viewed as less likely to receive messages about PrEP even though they could be appropriate candidates. In Philadelphia, it was noted that trans women engaged in sex work may not be willing to participate in spur of the moment outreach efforts by public health professionals about safer sex practices and PrEP use while working. This may be especially true if messaging comes from individuals unlike those most at risk. One participant (FG 1) said: “*When you’re out on the stroll you’re not gonna stop doing what you’re doing, making money, to sit there and talk to somebody about PrEP”.*

Participants from both locations shared multiple suggestions for ways that they believed PrEP could best be marketed to a diverse group of trans women. In Philadelphia, discussions arose regarding the importance of crafting messaging in accessible language and the importance of reaching those who may be hard to reach because they do not identify as part of the trans community. In Sacramento, participants talked about the assumptions that are made about trans women and that these types of assumptions are usually incorrect. One participant (FG 5) said, “*What really bothers me and turns me off about most advertising aimed at LGBT folks is the assumptions that are made- they just assume that when we see the word sex we’re talking about sex between the viewer who is either a cis-male or a trans-woman and someone with a penis”.*

In both locations, participants stressed that health messaging about PrEP may be best received if it is delivered by community members that resemble and share in the lived experiences of the intended target audience. A Philadelphia participant (FG 2) described the importance of including those from the trans community, especially young trans women of color: “*So we have to go to the cultures where they are, right … If you want people to go out of their way to come to you, has to be a reason for it … so … find some trans youth, pay them a little bit of money, go and teach them to do a lecture, take a doctor along with them and go in and teach some stuff and come away. Not somebody from a big organization. Somebody from the street, right? Um, somebody who’s a trans woman of color”.*

## Discussion

With PrEP scale-up quickly increasing and injectable forms on the near horizon [38], there is demand for culturally appropriate trans-specific messages in HIV prevention interventions and educational materials. Without acknowledging specific barriers to PrEP uptake among trans women and incorporating gender affirmation into PrEP education, simply knowing PrEP is available may not motivate trans women to use PrEP. In addition, our participants voiced dismay at current marketing efforts which problematically groups trans women alongside men who have sex with men. This grouping was perceived as inaccurate and may hinder PrEP uptake among trans women because effective PrEP marketing is foundational to improving usage.

Some interventions to increase PrEP uptake in trans women focus on addressing structural barriers [39, 40]. This is important as these barriers were echoed by our participants, specifically the lack of PrEP availability in trans inclusive healthcare setting. However, other knowledge gaps about PrEP in the trans women community exist. We found that there was confusion about the differences between PrEP and PEP and whether resistance to PrEP could occur if you took it intermittently. These continued knowledge gaps were also found in a recent study with trans women in New York and Washington DC [41]. Thus, this study expands a growing body of literature that stresses the need for increasing PrEP awareness among trans women, the importance of addressing trans-specific barriers to and concerns about PrEP use and underscores the necessity of working in collaboration with communities to optimize the messaging and delivery of information about PrEP to trans women.

There were some differences in knowledge, attitudes and use of PrEP in the two locations. Philadelphia participants were more likely to have used PrEP and had higher knowledge of both PrEP and PEP, while Sacramento participants expressed more concerns about hormone interactions. Philadelphia participants expressed more concern about HIV stigma and its relationship to PrEP, an issue that was not brought up in Sacramento. Philadelphia participants were also more likely to discuss how important being part of a trans women community was to having social support and to having important conversations about PrEP. Interestingly, the locations for these focus groups are both areas that have significant community and healthcare resources available for transgender individuals but none of the Sacramento participants had ever been on PrEP and discussed more overall barriers and misconceptions about PrEP use. One explanation for these differences is that the sample in Sacramento was younger and more likely to be Latinx, which may affect their engagement with trans-specific resources or healthcare. A recent study with Black and Latinx trans women on PrEP in Los Angeles noted that cultural or language barriers are often experienced that affect ability to use or access PrEP, especially when engaging with the healthcare system [42]. The Philadelphia trans women community, on the other hand, is reflective of the city’s demographics where over 50% are racial and ethnic minorities [43]. As a result, it may be that cultural and language barriers are not as prevalent in this location and may explain some of the differences in PrEP use seen between locations. These findings show that more understanding of regional conceptions about PrEP may be important to consider when developing PrEP uptake interventions and communication and further research in other parts of the country, especially areas that do not have trans-friendly resources, is needed.

In general, substantive research on how to interpret and address barriers to PrEP use among trans women must be prioritized. Results of this study indicate that important areas for future research include how to best provide trans-specific education and messaging about PrEP that ensures that trans women know about recent evidence of the safety of using PrEP and hormones [44] and addressing misinformation about PrEP use contributing to drug resistance. But communication is the proverbial “two-way street” and both providers and public health practitioners working with trans women need to ensure they are communicating not only the right messages but in the right context. Providers will need to better discuss the benefits of PrEP by addressing the concerns trans women may have in how it could potentially affect their transition. Additionally, providers may need to bring up PrEP in more than one session to help patients build comfort with addressing sexuality and gender, and to lessen feelings of mistrust. It is also important for them to stress the importance of daily adherence with their patients to optimize PrEP effectiveness and its minimal impact on hormone use.

Above all, it is important that educational messages and materials about PrEP are specifically geared to address trans women’s concerns, crafted in language that is widely accessible and used in interventions. Our participants emphasized how important it is to be provided PrEP information from respected community members and that a “one size fits all” approach will not work – this is especially pertinent for trans women of color who expressed the importance of racial representation in PrEP messaging. Recruiting trans women from a variety of identities who can speak from experience about PrEP will be essential in future communication interventions aimed at increasing knowledge and eventual uptake of PrEP. These community advocates can be an important bridge to healthcare, boosting confidence in asking for and discussing PrEP use with healthcare providers.

### Limitations

Limitations to this study include the location and time period, representativeness of trans women, and scope. Because focus groups were only done in large urban centers on the East and West Coasts of the United States, results may not represent knowledge and opinions of trans women in less urban areas or areas that do not have significant trans friendly resources. We also did not limit participation by PrEP status, which may have affected overall HIV risk perception. Despite this, we did see differences in knowledge of PrEP, perceptions about the benefits of PrEP, and actual use of PrEP. In addition, this study occurred in 2017 and perceptions of PrEP may have changed since then. However, recent literature indicates there is still poor understanding of PrEP in trans women [41]. Participants also represented a wide age range and ethnic makeup, indicating that the study did capture a variety of different voices. Future work should replicate this qualitative assessment in other parts of the country to further elucidate understanding of PrEP across different trans women communities.

## Conclusions

PrEP is a significant advancement in HIV prevention but many at-risk groups, including trans women, have not had optimal uptake and adherence. Understanding how PrEP is conceived, and how best to communicate the benefits of PrEP to trans women is critical to ensure they receive culturally appropriate messages that resonate with and address their needs.

## Supplementary Information

**Additional file 1.** Sociodemographic survey.

**Additional file 2.** Focus group moderators guide.

**Additional file 3.** Demographic characteristics by focus group.

**Additional file 4.** Sample quotes by focus group themes and sub themes.

## Data Availability

The data analyzed during the current study are available from the corresponding author on reasonable request.

## Abbreviations

HIV: Human immunodeficiency virus
PrEP: Pre-exposure prophylaxis
MSM: Men who have sex with men
FG: Focus group
