# Supplementary material for: Exploring barriers and facilitators to PrEP use among transgender women in two urban areas: implications for messaging and communication
Source: BMC Public Health. 2022 Jan 6;22:17. doi: 10.1186/s12889-021-12425-w (PMC8740429; doi:10.1186/s12889-021-12425-w)
Supplement: Supplementary file 1 — Additional file 1. Sociodemographic survey. [file 12889_2021_12425_MOESM1_ESM.pdf]

DEMOGRAPHIC SURVEY – Focus Groups

Focus group # _______

Following are some questions to help us understand who is participating in the focus groups. Please mark the box that best describes you. Thanks!

1. What is your current gender identity? ***(Check all that apply.)***

- ❑ Male (1)
- ❑ Female (2)
- ❑ Transgender Male (3)
- ❑ Transgender Female (4)
- ❑ Genderqueer (5)
- ❑ Additional Category (specify): (6)
- ❑ Decline to Answer (99)

2. What sex were you assigned at birth? ***(Select one.)***

- ❑ Male (1)
- ❑ Female (2)
- ❑ Decline to Answer (99)

3. What is your race/ethnicity? ***(Check all that apply.)***

- ❑ African American/Black (1)
- ❑ Latino(a) (specify ethnicity): (2)
- ❑ Pacific Islander (specify ethnicity): (3)
- ❑ Asian (specify ethnicity): (4)
- ❑ Native American or Alaska Native (5)
- ❑ White/Caucasian (6)
- ❑ Multiracial/multiethnic (specify ethnicities):(7)
- ❑ Other (specify): (8)
- ❑ Don't Know (98)
- ❑ Decline to Answer (99)

4. How old are you now? ***(Select one.)***

- ❑ _________ years old (1)
- ❑ Don’t Know (98)
- ❑ Decline to Answer (99)

5. What is the highest level of school you finished? ***(Select one.)***

- ❑ Less than high school. Highest grade of school completed: (1)
- ❑ Finished high school or got GED (2)
- ❑ Technical or vocational school or community college (3)
- ❑ Some college (4)
- ❑ College degree or above (5)
- ❑ Don't Know (98)
- ❑ Decline to Answer (99)

6. In the past 6 months, what were your sources of income and financial support? *(Check all that apply.)*

- ❑ Employed full-time job (1)
- ❑ Employed part-time (2)
- ❑ Employed sometimes (3)
- ❑ Government Assistance (AFDC, food stamps, etc.) (4)
- ❑ Disability (5)
- ❑ Unemployment benefits (6)
- ❑ VA Benefits (7)
- ❑ Social Security Insurance (SSI) (8)
- ❑ Sex for pay (prostitution) (9)
- ❑ Spouse/partner provides income (10)
- ❑ Other family member(s) or friends provide income (11)
- ❑ Selling drugs (12)
- ❑ Alimony or child support (13)
- ❑ Scamming/stealing (14)
- ❑ Other (specify): ______________________________ (15)
- ❑ Don't Know (98)
- ❑ Decline to Answer (99)

7. In the past 30 days, how much money have you received from all sources? *(Select one.)*

- ❑ $0 - $500 (1)
- ❑ $501 - $1000 (2)
- ❑ $1001 - $2000 (3)
- ❑ $2001 - $3000 (4)
- ❑ $3001 - $4000 (5)
- ❑ $4000 or more (6)
- ❑ Don't know (98)
- ❑ Decline to Answer (99)

8. Which of the following statements best describes your financial situation: *(Select one.)*

- ❑ I have enough money to live comfortably. (1)
- ❑ I can barely get by on the money I have. (2)
- ❑ I cannot get by on the money I have. (3)

9. On a scale from 1 to 10, with 1 being not at all sure and 10 being totally sure, how sure are you that you will be in the same home a year from now? *(Circle one.)*

1 2 3 4 5 6 7 8 9 10

10. During the past 6 months, where did you live most of the time? *(Select one.)*

- ❑ A house or apartment you own (1)
- ❑ A house or apartment you rent (2)
- ❑ Live with main partner (3)
- ❑ Live with friends or family (4)
- ❑ A halfway house or treatment center (5)
- ❑ Someone else's house or apartment (6)
- ❑ A shelter (7)
- ❑ A motel, hotel, boarding house, or SRO (8)
- ❑ On the street or in a park, abandoned building, car, etc.(9)
- ❑ Other (specify): ______________________________ (10)
- ❑ Don't Know (98)
- ❑ Decline to Answer (99)

11. Have you <u>ever</u> been homeless or lived in a shelter? ***(Select one.)***

- ❑ No (1)
- ❑ Yes (2)
- ❑ Don't Know (98)
- ❑ Decline to Answer (99)

<u>IF YES</u>, when was the last time you were homeless or staying in a shelter? (Select one.)

- ❑ In the past month (1)
- ❑ Between one month and six months ago (2)
- ❑ Between six and 12 months ago (3)
- ❑ Longer than a year ago (4)
- ❑ Don't Know (98)
- ❑ Decline to Answer (99)

13. What is your current ZIP code? ***(Select one.)***

- ❑ ____________________ (1)
- ❑ Don't Know (98)
- ❑ Decline to Answer (99)

14. What kind of health insurance do you have now? ***(Check all that apply.)***

- ❑ Medicaid (1)
- ❑ Medicare (2)
- ❑ Veteran's Administration (3)
- ❑ Private Insurance or HMO (4)
- ❑ None (5)
- ❑ Other _________(6)
- ❑ Don't know (98)
- ❑ Decline to Answer (99)

15. Have you ever heard about PrEP (pre exposure prophylaxis medication) from a doctor or other healthcare provider? ***(Select one.)***

- ❑ No (1)
- ❑ Yes (2)
- ❑ Don't know (98)

16. Have you ever heard about PrEP from a family member, friend or someone in your community? ***(Select one.)***

- ❑ No (1)
- ❑ Yes (2)
- ❑ Don't know (98)

17. Have you ever used PrEP (pre-exposure prophylaxis medication)? ***(Select one.)***

- ❑ Yes (1)
- ❑ No (2)
- ❑ Not sure (98)

<u>IF YES</u>, how long did you take it? ***(Select one.)***

- ❑ One month or less (1)
- ❑ 1-3 months (2)
- ❑ 3-6 months (3)
- ❑ 6 to 12 months (4)
- ❑ I am still on it (5)
- ❑ Don't know (98)
