# Supplementary material for: Exploring barriers and facilitators to PrEP use among transgender women in two urban areas: implications for messaging and communication
Source: BMC Public Health. 2022 Jan 6;22:17. doi: 10.1186/s12889-021-12425-w (PMC8740429; doi:10.1186/s12889-021-12425-w)
Supplement: Supplementary file 2 — Additional file 2. Focus group moderators guide. [file 12889_2021_12425_MOESM2_ESM.pdf]

<u>**Focus Group Guide**</u>

Study Title: Mapping Perceptions of PrEP use in Trans Women and Barriers to Care in Providers to Inform Intervention Development

<u>Focus group number:</u>

<u>Date:</u>

<u>Facilitator Initials:</u>

(NOTE TO FACILITATOR: Read only bolded text. Additional text is provided to prompt. We ideas for probes, so read only as needed to guide the conversation.)

Recent studies have found that using antiretroviral medication (the same medications that HIV-positive people take) before being exposed to HIV was beneficial in protecting people from getting HIV. This approach is known as PrEP, which stands for Pre-Exposure Prophylaxis. The purpose of this focus group is to find out what transgender women think about the idea of PrEP, whether it is something you would be interested in and what concerns you might have about it.

By offering verbal consent, you have agreed to participate in a focus group to discuss this topic. We thank you in advance for your participation. Your thoughts are important for us to understand whether PrEP could work as an HIV prevention option for transgender women.

<u>Discussion format:</u>
We would like the discussion to be informal, so there's no need to wait for us to call on you to respond. In fact, we encourage you to respond directly to the comments other people make. Please speak one at a time so we can all hear what is being said. If you don't understand a question, please let us know. We are here to ask questions, listen and make sure everyone has a chance to share. There are no wrong or right answers. As you saw in the consent, we will be audio recording the discussion, because we don't want to miss any of your comments. No one outside of this room will have access to these recordings and they will be destroyed after our report is written.

<u>Introductions:</u>
(*Introduce yourself and your co-facilitator.)* **I'm going to ask each of you to introduce yourself to the group. Please only give a first name. Also the name you choose to introduce yourself does not need to be the name you usually use. You can introduce yourself using any first name you choose.** *(Have participants introduce themselves by their first name only.)*

<u>Ground rules:</u>
Now I want to go over a few guidelines for the group discussion.
1. What we say in the group should stay here – keep it confidential.
2. One person talks at a time. Because we would like to hear from everyone, I might call on you if you're not saying much, or ask you to give others a chance if you have talked a lot.
3. Feel free to respond to another group member, not just to my questions. You can follow up on what someone has said, agree or disagree, or give an example. Please speak for yourself using "I" statements and avoid attacking or putting someone else's ideas down.

4. We'll be talking some about sex, which can be a sensitive topic. It's OK to talk about anything at all here. We really want to hear from you. If you prefer, you may talk about trans women in general instead of your own experiences if you would like.
5. We want to hear a lot of different perspectives, so as a group we have to respect everyone's point of view. Everyone won't agree, but we want to hear from everybody. There are no wrong answers! We want to ensure a safe environment where individuals speak openly so remember that everyone's opinion and experience is valuable.
6. To protect everyone's privacy, please use only the first names for everyone here.
7. Feel free to get up from the table at any time. You may want to get more snacks, go to the restroom or just take a break or a stretch.
8. Please take a moment now to turn off all cell phones or place them on silent. (Give them a moment now to do this.)
Are there any ground rules that we should add to these? (Give them a moment to discuss.)
Can everybody work with these guidelines? (Allow them to confirm.)
Okay, let's begin. I will turn on the digital recorders now.
(TURN ON DIGITAL RECORDERS.)

<u>Introductions/ Ice Breaker:</u>

I'm going to ask each of you to introduce yourself to the group. Please only give a first name. Also the name you choose to introduce yourself does not need to be the name you usually use. You can introduce yourself using any first name you choose. Please also let us know where your name came from and what special meaning it has.

<u>PrEP knowledge</u>

1. **To begin our conversation, what have you heard about PrEP?**

Potential probes:

- Are people you know talking about PrEP? If so, what are they saying?
- Have you known anyone who has taken PrEP? What was their experience?
- Have you or anyone you know ever been offered PrEP?

2. **Let's talk a little more about the facts about PrEP. Is anyone on PrEP now or has been in the past and is willing to share their thoughts?**

Here is how PrEP works. People who are HIV-negative can take a prescription medication (tenofovir, also known as Truvada), in order to prevent becoming infected with HIV. To be effective, the medication must be taken every day. If you don't take the pill every day, it is not as effective in protecting against HIV.

3. **What do you think about this strategy for HIV prevention? OR What are the initial things that come up for you when you think about using it yourself?**

Responses will likely correspond to one of the 4 domains below – pursue questions within each domain.

Adherence	Side Effects	Social Issues	Sex
- What do people think about taking a pill every day?<br>- Does that sound like something that could work for you?<br>- What would it take for you to work it into a daily routine?<br>- What might get in the way of talking it daily?<br>i.e. forgetting, concerns about disclosing PrEP use to sexual partners or others, drug or alcohol use, HIV-related stigma	Studies have found that very few people experience side effects while taking PrEP.<br><br>What are your specific concerns about side effects that would influence your decision to take PrEP?	What other barriers to using PrEP might impact you and your transfriends?<br><br>What concerns might you have if you're a sex worker or you use substances?<br><br>Would you have any concerns about your sexual partners and/or friends finding out that you were taking PrEP? Why or why not?	Do you think taking PrEP would increase or decrease your sexual satisfaction?<br><br>In what ways would using PrEP influence condom use among you and your transfrriends?<br><br>What about if you were a sex worker?<br><br>Do you think that taking PrEP would change the decisions you make about sex? If so, how? If not, why not?

<u>Care seeking, Appointments, Hormones</u>

4. In order to get PrEP, you have to ask a doctor to write you a prescription for it. How comfortable would you and/or your transfriends feel asking their medical provider/doctor for PrEP?

Potential probes:

- Do you think trans women in your community are generally comfortable talking to their doctors about sex? Why or why not?

- Do you think you would also take PrEP while getting your hormones?
- Do you have concerns about PrEP's interaction with hormones?

5. While taking PrEP, people need to see their doctor once every 3 months. Would you and/or your transfriends be willing to go to medical appointment once every 3 months in order to use PrEP? Why or why not?

- What do you think about your ability and trans women in your community's abilities to make and keep quarterly doctor's appointments?
- Where would you go to seek PrEP?

<u>HIV Testing</u>

Before you start using PrEP it is important to get tested to be sure you are HIV-negative. When taking PrEP, it is recommended that people get tested every 3 months.

6. Do you think that your and/or your trans friends would be willing to get tested every 3 months in order to use PrEP? Why or why not?

- How often do you and your trans friends normally get tested for HIV? Where do they go to get tested?

<u>Cost</u>

7. What do you think people need to know when it comes to the costs associated with taking PrEP?

Potential probes:

- What questions comes to mind when it comes to the cost of PrEP?
- Are you aware of the fact that PrEP can usually be provided to you for free?

Potentially mention:
Because PrEP requires a prescription and medical appointments, some people might think it is expensive or out of reach because they do not have health insurance. However, many programs have staff called PrEP navigators that help people sign up for insurance. With insurance, many people can get PrEP for free or very low cost.

Concluding thoughts

When you think about whether taking PrEP would be right for you, what are the biggest factors that influence your thoughts about this?

- Probe both pros and cons: cost, side effects, adherence issues (i.e. remembering to take a pill every day, keeping regular doctor's appointments), stigma (i.e. people thinking the person is HIV-positive)
- Do you think that trans women in your community would be interested in taking PrEP? What do you think would make them more likely to use PrEP?

Thank you very much for your time today. I would like to remind everyone that we have asked for everything that has been said today to be kept confidential. The information you have provided will be very helpful in helping us to think about the right HIV prevention methods for transgender women.
