# Supplementary material for: Exploring barriers and facilitators to PrEP use among transgender women in two urban areas: implications for messaging and communication
Source: BMC Public Health. 2022 Jan 6;22:17. doi: 10.1186/s12889-021-12425-w (PMC8740429; doi:10.1186/s12889-021-12425-w)
Supplement: Supplementary file 3 — Additional file 3. Demographic characteristics by focus group. [file 12889_2021_12425_MOESM3_ESM.docx]

| **Characteristic** | **FG 1 (n=10)** | **FG 2 (n=3)** | **FG 3 (n=7)** | **FG 4 (n=8)** | **FG 5 (n=6)** | **Total (N=34)** |
| --- | --- | --- | --- | --- | --- | --- |
| **Age**, mean (SD)^ǂ^ | 50.2 (30) | 52.33 (15) | 39.57 (27.41) | 29.63 (9.99) | 40.17 (13.62) | 36.03 (14.3) |
| **Race,** N(%)* | | | | | | |
| African American | 4 (40) | 0 (0) | 4 (57.1) | 5 (62.5) | 2 (33.3) | 15 (44) |
| White | 3 (30) | 2 (66.7) | 3 (42.9) | 2 (25.0) | 4 (66.7) | 14 (41) |
| Latinx | 1 (10) | 0 | 0 (0) | 4 (50) | 2 (33.3) | 7 (21) |
| Asian | 0 (0) | 0 (0) | 1 (14.3) | 0 (0) | 0 (0) | 1 (2.9) |
| Native American or Alaska Native | 0 (0) | 1 (33.3) | 0 (0) | 1 (12.5) | 0 (0) | 2 (5.9) |
| Another race | 1 (10) | 0 (0) | 0 (0) | 0 (0) | 0 (0) | 1 (2.9) |
| Declined | 1 (10) | 0 (0) | 0 (0) | 0 (0) | 0 (0) | 1 (2.9) |
| **Gender Identify**, N(%)* | | | | | | |
| Female | 1 (10) | 0 (0) | 1 (14.3) | 3 (37.5) | 2 (33.3) | 7 (21) |
| Transgender | 9 (90) | 3 (100) | 6 (85.7) | 5 (62.5) | 5 (83.3) | 28 (82) |
| Queer | 0 (0) | 1 (33.3) | 0 (0) | 0 (0) | 1 (16.7) | 2 (6) |
| Additional | 0 (0) | 0 (0) | 0 (0) | 0 (0) | 1 (16.7) | 1 (2.9) |
| **Educational entertainment**, N(%) | | | | | | |
| High school and below | 5 (50) | 1 (33.3) | 5 (71.4) | 3 (37.5) | 1 (16.7) | 15 (44) |
| Some college and above | 5 (50) | 2 (66.7) | 2 (28.6) | 5 (62.5) | 5 (83.3) | 19 (56) |
| **Homelessness experienced**, N (%) | 6 (60) | 2 (66.7) | 4 (57.1) | 5 (62.5) | 3 (50) | 20 (63) |
| ^ǂ^ Includes only people who disclosed their age (n=31)  * Category is not exclusive | | | | | | |
