# Supplementary material for: Exploring barriers and facilitators to PrEP use among transgender women in two urban areas: implications for messaging and communication
Source: BMC Public Health. 2022 Jan 6;22:17. doi: 10.1186/s12889-021-12425-w (PMC8740429; doi:10.1186/s12889-021-12425-w)
Supplement: Supplementary file 4 — Additional file 4. Sample quotes by focus group themes and sub themes. [file 12889_2021_12425_MOESM4_ESM.docx]

**Table 3. Sample Quotes by Focus Group Themes and Sub-topics**

| **Theme** | **Sub-topics** | **Quotes** |
| --- | --- | --- |
| **Continued lack of knowledge about PrEP and its use** | PrEP knowledge and effectiveness | What PrEP does, what Truvada does, what all of the antiretrovirals do is stop it [HIV] from getting into the cell in the first place. *Sacramento FG 5*  Truvada doesn't do anything to AIDS. It's HIV. And it's two completely different things. For folks living with HIV it's real hurtful when they hear somebody like confusing AIDS for HIV. *Sacramento FG 4*  What I was talking about earlier with how, how bad this city is with letting everybody know how bad the epidemic is right now with HIV and syphilis and they're not talking about that, which is serious, why would they talk about PrEP, right? There's really no education out there and it's not something that's actively on anybody's mind. *Philadelphia FG 2*  It's a HIV pill that you take every day and to like help you from getting the HIV and it does help you along, the PrEP, you can't skip a dose cause that gonna fuck with your system. *Philadelphia FG 3*  It is much more effective. It has the highest effectiveness ratings for anal sex involving penises. Um, in fact, if you start PrEP within 7 days, you are over 90% protected rectally. *Sacramento FG 5*  Yeah I also heard that if taken every day, it has a really high rate of effectiveness in clinical studies comparable to the use of a condom, so it provides another option or another layer of protection for folks who are having unprotected sex, or protected sex. *Sacramento FG 5*  There are still drug resistant strains [of HIV] out there that are immune to PrEP, that you cannot even treat with PrEP, so there's nothing that can be done for those. *Sacramento FG 4*  I know with the statistics it’s think it’s 99.9% effective or something like that. *Philadelphia FG 1*  I think it does reduce you getting HIV, I think it's like 92, is it 93 or 92 percent. Up to 92% I think it is? Yeah but it's so that you still have that chance of still getting it, so, yeah. *Philadelphia FG 3* |
|  | PrEP resistance | I had some reservations about PrEP, mainly the resistance that this young lady talked about. I didn't want to become resistant to something that I may in the future may need, you know. There's always a chance that I may catch the virus even if I take PrEP. *Philadelphia FG 3* |
|  | STI (Sexually Transmitted Infectious Diseases) Non-protection | It prevents HIV but it doesn't prevent STDs. *Sacramento FG 4*  It can help prevent, you know, that virus [HIV], even though there's many other viruses and STIs that you can catch out here, at least one of the biggest ones is under control. Not to say that it's even under control, just to say that they got something that's targeting that specific STI. *Philadelphia FG 3*  It's really an awesome thing this PrEP, an awesome drug, but it's something people still have to be vigilant, and still practice safe sex. *Philadelphia FG 3* |
|  | Difference between PEP and PrEP | A friend of mine, we had talked about PrEP before. I know he said that he was taking it and I had um, been exposed to the disease itself before so it's something that I had took but I guess it was PEP and not PrEP. *Sacramento FG 4*  When I took the PEP, I felt a difference in my body at the time. I felt nauseous. I felt fatigue. I felt like just not there, like spaced out sometimes and I kinda just wanted to be at home. And there were days I felt the pain in my side. *Sacramento FG 4*  Yeah, in my case I was exposed to the HIV so I felt what the doctor suggested was to take the PEP but that's something different from the PrEP*.  Sacramento FG 4*  Doesn't ring a bell. Sacramento FG 4  I never heard of PEP. What was that? *Sacramento FG 4*  That's what PEP is for. That's what I'm saying like if you know you have a problem, go solve  your problem. It's cheaper that way so I'd do that one instead of doing some regularly thing every day, damaging my kidneys and not being able to afford it anyway. *Sacramento FG 4*  It's [PEP] a much riskier alternative, because you don't know, you don't know how much you're already effected, or infected. *Sacramento FG 4*  No PEP is, PEP is different. PEP is something, if you were already exposed to HIV then your doctor probably would prescribe you PEP just to make sure that you don’t contract it or something of that nature. *Philadelphia FG 1*  I just knew like as far as like  have felt that you have come into uh, you know, had sex with someone that is HIV positive, they encourage you, get into, take PEP. *Philadelphia FG 3*  I personally have had experiences with both PrEP and PEP and the way they did it for me was they did, they when I came in saying that I had, it was a possibility I had been exposed. They did bloodwork and immediately put me on PEP and it's basically a 28 day regimen. *Philadelphia FG 3*  Post exposure, it's typically reserved for those, um, like…rape victims and children born with HIV and it's a very aggressive cocktail of HIV anti-virals, um, medication. That's what I heard. *Philadelphia FG 3* |
| **Persistent structural and personal barriers to PrEP use** | Pill taking | I wouldn’t mind taking it if it wasn’t harmful. I’d take it everyday. Why not? Like a vitamin. *Sacramento FG 4*  When you go from not used to having to take a pill everyday, to relying on a pill everyday- a girl like me can’t even dedicate herself to taking her hormone shots every week, let alone having to take a pill everyday. *Philadelphia FG 3*  One of the things that was a big challenge for me was taking the pills every day. But I’m also on HRT so it kinda really fits into the balance, keeps me grounded. I have to be mindful though like if I go away from my home for a couple nights, I have to remind me to take the pill with me. *Philadelphia FG 2*  ...It’s about getting on a schedule. *Philadelphia FG 3* |
|  | HIV Stigma and PrEP | On the off chance that I've had to reveal to people that I am on PrEP, which I am, that the first thing they do is like "do you have HIV"? I'm like "No, key word: prevention.” It's kind of annoying to me that people, when they hear  something even the word HIV in it they automatically think that you're positive. *Philadelphia, FG 3*  There's a stigma attached to us just in general as a community...what you see especially in our own community, is that unless a girl is physically murdered, she died of AIDS… because children is quick to any notion that they trampy and charge it to complications [of HIV]. I done know girls personally who have passed away from other health related issues. Wasn't even positive. But when people that don't "Oh child, you know she had that you know woo woo” and all of that stuff. The stigma as a community, it's already attached to us, already whether you think so or not or whether you feel like maybe the minute something happened, they all think that we all HIV positive already. *Philadelphia FG 3*  I'm like, "you know about PrEP?" so, you know, "What's PrEP?" I said. The first thing he asked me was "You got that shit?" I'm like "what no, read what it says." So, once once, you know, he start reading he's like "Oh, I got you, I got you". *Philadelphia FG 3* |
|  | Healthcare Experiences | If you’re not on the best most personal terms with your doctor, like just bringing up anything sexually related could be sort of a chore. *Sacramento FG 5*  I personally think that having to go through a doctor as a gatekeeper would be a pretty significant barrier for a lot of trans women and femmes because were already really marginalized in most medical environments- I mean most healthcare workers are not trained on trans-sensitivity or trans cultural competence or structural competence. They are mostly pretty clueless and often misgender us or our bodies in a careless or even deliberate manner. *Sacramento FG 5*  So my point is there’s a lot of- I feel a lot of hesitation, distrust when it comes to healthcare providers. I’m sure that if I were currently doing survival sex um or sex work, if I were a trans woman of color I would have all the more reason to be distrustful. *Sacramento FG 5*  It was me who, you know, had seen the poster and called up, you know, so it wasn't something that my doctor actually, you know. All for it once I brought it up, but it was like, you know… *Philadelphia FG 2*  I've seen doctors but nobody's ever given me the option of going on PrEP. *Philadelphia FG 2*  Doctors have to be able to authorize and actually maybe even suggest it, if you're a trans woman, suggest giving you the option to do it. I didn't hear anybody say anything. *Philadelphia FG 2*  I don't think it's really widely discussed even from medical providers here unless you bring it up. *Philadelphia FG 2*  *When I didn't live in Philadelphia, the health care providers were even worse, alright. So, you go to a doctor and you get a medical exam from the doctor in the doorway telling you to do self-exams cause they're afraid to walk in the room just cause you're trans. Philadelphia FG 2*  *A lot of people don't know about it, alright, and although I can tell them about it right, they still have to find a doctor that can, that can prescribe it, knows enough about it to prescribe it to them. Um, there's a stigma that if you're taking it, that you're dealing with somebody who's HIV positive, or your engaging in sex work. Philadelphia FG 2* |
| **Beliefs about how PrEP should be integrated into sexual behavior** | Who should take PrEP | The way I look at it. If I was gonna be out with somebody I didn't know. Hell yeah I'd be taking it for three weeks before I go meet that person. *Sacramento FG 4*  If you're married, if you're not necessarily having high risk sex, it may not be as necessary as if you are having high risk... If you have a monogamous relationship or you're not ill where there's any chance of that, I mean it's something to have on the side, but I don't think it's something that, personally, I think that I would want to take on a day to day basis, unless those factors were involved. *Sacramento FG 4*  Even though I’m polyamorous, I’m just seeing one person right now, and we know each other pretty well and I know that she’s HIV negative so I’m not really worried about becoming positive through that relationship. But I think if my circumstances changed in some way I might be. *Sacramento FG 5*  If I was going to go out and just start going to San Francisco every weekend, going to different places and just having a lot of hookup/anonymous sex, yeah, that’s probably a pretty appropriate place to do it - or to be taking PrEP. *Sacramento FG 5*  My younger sister, I explained to her the other day she was telling me she kind of still has an active sex life. I feel me personally, I think well it would be good for you. You know? My other sister, she’s not very sexually active to my understanding, so I really don’t promote it as much to her because I mean the idea is HIV and I think normally people identify HIV with sexual intercourse. You know? They don’t take into consideration that other actions can cause that problem as well. *Sacramento FG 5*  In my case, my partner has HIV so I would take it so I would be safe having sex with him. *Sacramento FG 5*  I would not classify myself as very sexually active. I just have one...it's just a friend with benefits and we don't have to answer to each other or nothing...I just wanted protection, is what it was, you know? And uh, I felt good about that. *Philadelphia FG 2*  I initially started it and then I got into a long-term relationship and at that point, we both established that we did not have HIV and I felt comfortable enough with the person to not use it anymore. *Philadelphia FG 3*  I was kind of closed minded to it cause I'm like, you know, I'm not at high-risk behavior so why would I even get on, and he was like  you know, you never know what your partner is doing. That's what ha-, that's what my mind was, damn, you're right, you, you never know what your partner is doing and it kind of encourage me to get on it just to, just to protect myself. *Philadelphia FG 3*  I am married to somebody so it was like, you know, I take it just to be on the safe side. You never know when somebody’s being dishonest to you so when I heard about it, it was actually a breakthrough but it was like ease when they told us about it*. Philadelphia FG 3* |
|  | Sex work | I think it would be helpful- it would have been helpful back then for me. I would have been excited, I probably would have been telling all my friends like “hey ladies! we’re going to be hookers this weekend, let’s go make sure were safe (er). *Sacramento FG 5*  I took it as long as I knew my risk factors were high when I was doing sex work and escorting and things like that. *Philadelphia FG 3* |
|  | Condom use | I like to have unprotected sex. Because it feels much better without having a condom on. Or for the person I'm having sex with. But I still make sure I'm clean though and that he's clean. Just by asking a question- like just say yes or no. Now if you say maybe then I feel like you're lying to me. So now I know you're HIV positive if you say ehhh iffy maybe. But, PrEP is a pretty good way to stay clean. *Sacramento FG 5*  If the clients know that the person they’re seeing that- the sex worker that they’re on a date with- has been taking PrEP, then they might use that as a way to pressure them not to use condoms. If the sex workers don't tell them, then they're still going to face a lot of pressure to not use a condom. *Sacramento FG 5*  I mean it might possibly lead to someone using condoms less, which like could be sort of uh, a negative sign. But I mean honestly using both in conjunction would be the safest. *Sacramento FG 5*  I get more people who demand, require, unprotected sex than protected sex. *Philadelphia FG 3*  I think people have replaced the condom with PrEP...a condom is not gonna protect you completely anyway against Herpes, genital warts, and things of that such. I don't know why people would think that that's a replacement, or even an adequate replacement but I know that people and disenfranchised communities which a lot of us are a part of or near, do not like using condoms anyway, so that was never even part of the equation to begin with. *Philadelphia FG 3*  I think people are jumping on the PrEP bandwagon because they feel like, 'I don't want to use condoms, so I'm going to use PrEP because some protection is better than none. *Philadelphia FG 3*  I think that's the misconception that since I'm on PrEP I ain't gotta use condoms no more. I think they forget that it's more than just HIV out there and I think that's a scary thing. *Philadelphia FG 3* |
| **The importance of the trans community in promoting and communicating about PrEP use** | Reaching the “hard to reach” | If someone like a sister came up to me like "Girl, like this is the T. Like I'm on it. You need it." I'd be more acceptable to trying it. *Sacramento FG 4*  Start talking about sex more. *Sacramento FG 5*  But there are people who do not walk the street. There are people who work from home...a great challenge is: how do you reach them. I don’t have the answer but I’m posing the question because it’s so important. They are the most at risk people. *Philadelphia FG 1*  The most at risk people should be targeted as the people to be reached...I think the most at risk people can sometimes be the most challenging people to reach. The girls on the corner. The girls in the bar. We can find them. *Philadelphia FG 1*  I think that when we are targeting high risk communities that the people that are targeting them are the people that look like them...to target someone that’s high risk, you either have to be somebody that’s high risk or have been in that situation and the girls from the stroll wouldn’t talk to me if I wasn’t a girl from the stroll. *Philadelphia FG 1* |
|  | Concerns and suggestions | Gay men knew about it [PrEP] six years ago. *Sacramento FG 4*  They promote smoking cigarettes. They promote the new tequila. They promote the new burgers. All these things that aren’t helpful to our health, but this is something that I have not seen on social media nor TV or anything, cable, nothing. *Philadelphia FG 1*  I haven’t seen anything on my television that was PrEP related, I mean, and I haven’t even seen nothing on social media unless it was [Community Health Center] or one of these orgs that, that was promoting it. *Philadelphia FG 1*  The New York Pride Parade was just Sunday. I don’t think I once saw a PrEP banner, a PrEP anything being passed out. *Philadelphia FG 1*  I know that a lot of folks, well trans folks, didn’t think that PrEP was for them because of the way that PrEP has been presented. They had the perception that PrEP was only for MSM. *Philadelphia FG 1*  They don’t have a trans category. They don’t recognize trans. They recognize men and women. *Philadelphia FG 1*  I'm actually like surprised at how like widespread it's become like, I'm actually seeing ads for PrEP in places I didn't think I'd see them. I'm seeing them out in Kensington. I'm seeing them, of all places on Grindr. *Philadelphia FG 3*  Look at the Tuskegee experience. When you have a white person, even black people, offering you something and trying to push, first of all I think that those providers need to not push PrEP like you know, trying to push it, but actually just care about people. If they don’t want the PREP, then oh well. Don’t try to work for the company or the grant or whatever. Work for the people. *Sacramento FG 4*  This young generation now-a-days, everything’s technology. Do some social reach, like social media and stuff. *Sacramento FG 4*  And I think that like place like this, the staff needs to reflect the communities that they want to reach. And I think they, if they wanna start helping young girls, institute some young girls in the staff. Sacramento FG 4  A lot of trans people are also IV drug users. I mean because we use it for muscular estrogen. For trans folks who are not like accessing that through prescriptions...they may go to alternate routes and means to obtain their injection estrogen and they end up sharing needles if they don't have access to clean ones. So I mean is that also something that’s being considered in marketing?  *Sacramento FG 5*  A lot of the information that’s shared to the community gets lost in translation. Sometimes it takes for someone to come and explain things in, using layman's terms so that people can understand what they’re saying. You know, when you use a bunch of clinical “blah blah blah” that’s exactly what it sounds like and then people leave without the knowledge that they actually came to get. *Philadelphia FG 1*  What I try to do is educate on a one-on-one basis. That’s how I do it. *Philadelphia FG 3* |
